# A new role for spinal manual therapy and for chiropractic? Part II: strengths and opportunities

**DOI:** 10.1186/s12998-024-00532-5

**Published:** 2024-03-27

**Authors:** Søren Francis Dyhrberg O’Neill, Casper Nim, Dave Newell, Charlotte Leboeuf-Yde

**Affiliations:** 1 Medical Spinal Research Unit, Lillebaelt Hospital, Spine Centre of Southern Denmark, University Hospital of Southern Denmark, Østre Hougvej 55, Middelfart, Denmark; 2 Department of Regional Health Research, University of Southern Denmark, Odense, Denmark; 3 Center for Muscle and Joint Health, Department of Sports Science and Clinical Biomechanics, University of Southern Denmark, Odense, Denmark; 4 AECC University College, Bournemouth, UK

**Keywords:** Chiropractic, Chiropractic History, Professional Identity, Professional Development, Spinal Manipulation

## Abstract

In a previous paper, we presented some important weaknesses of and threats to the chiropractic profession as we see them. We further argued that the chiropractic profession’s relationship with its principal clinical tool (spinal manual therapy) is at the core of the ideological divide that fractures the profession and prevents professional development towards greater integration in the healthcare landscape. In this manuscript, we shall argue that the historical predilection for spinal manipulation also gifts the profession with some obvious strengths and opportunities, and that these are inextricably linked to the management of musculoskeletal disorders. The onus is now on the chiropractic profession itself to redefine its raison d’être in a way that plays to those strengths and delivers in terms of the needs of patients and the wider healthcare system/market. We suggest chiropractors embrace and cultivate a role as coordinators of long-term and broad-focused management of musculoskeletal disorders. We make specific recommendations about how the profession, from individual clinicians to political organizations, can promote such a development.

## Background

### Spinal manipulation and the chiropractic profession

In a previous paper we outlined the threats and weaknesses that arise from the chiropractic profession’s unique relation to spinal manual therapy (SMT), which we believe places the chiropractic profession in a most precarious position. Despite the obvious relevance of chiropractic care for musculoskeletal (MSK) disorders, many chiropractors seem to devote their attention towards non-MSK conditions and *specialize* in other obscure areas, such as maximizing infants’ development, curing brain injuries, or improving athletic performance. Others overemphasize the role of SMT in the treatment of MSK conditions, with high expectations of its curative and preventative powers. In general, there is also an undue emphasis on the technical specifics of SMT, which it seems that many chiropractors expect to be the key to solving the patient’s problem.

We went on to speculate that, for these reasons, from an international perspective, chiropractors may have already missed the boat as far as establishing cultural and formal authority in the MSK arena. We also contended that there are countries where this is still possible but by no means easy.

In this follow-up paper, we shall attempt to identify and describe aspects of the use of SMT that constitute significant strengths and opportunities for the chiropractic profession.

On that basis, the central aim of this paper is to articulate our belief that the chiropractic profession can and must re-invent itself with some urgency and with a critical focus on re-assessing the role of SMT. This entails a shift in chiropractic identity away from that of providers of SMT within a separate and distinct theoretical framework towards a broader role as expert patient-centred care providers and coordinators of long-term management of MSK disorders in the wider healthcare system.

## Strengths

### Safe, low cost, available

Irrespective of which side of the professional divide chiropractors come down on, the clinical reality, as it will appear to an external observer, is that chiropractors generally provide care, which is safe, low-cost, low-tech, accessible, engaging, conservative, and often focused on long-term management. Such an approach seems particularly pertinent for MSK problems, which are often chronic or recurring, and SMT can play an important role in that clinical approach.

Against the background of the potential negative consequences of other commonly used treatments for MSK disorders, such as opioids and surgical procedures, the safety and low-cost availability of SMT are obvious strengths. Thus, Coulter et al. [1] argue that there are compelling and ethical arguments why medical health-care providers should reach out to consult a chiropractor and consider non-pharmacological treatments such as SMT for musculoskeletal pain.

Whilst factors such as safety, availability, and cost are undoubtedly important, they are not the only strengths of SMT.

### Contextual factors of SMT

Contextual factors, which generally consist of cognitive cues embedded in therapeutic encounters [2], are becoming increasingly evidenced as important for positive clinical outcomes, both in health-care in general and in MSK disorders specifically [3].

More than anything, MSK disorders are characterized by pain, and many patients seek out chiropractic care for acute pain episodes. Anecdotal and scientific evidence suggests that SMT can have an immediate pain relieving effect on MSK pain [4–7]. We suggest that SMT also provides a unique, often advantageous contextual frame to the continued clinical management of MSK disorders:

The contextual frame of SMT is one where patients with acute MSK pain will find themselves in the office of a chiropractor that can (a) literally ‘point out’ the source of the pain by putting their finger on it (e.g., palpating tender tissues), (b) offer an explanation which ties together the symptoms with the treatment provided and, as an added bonus, also explains future recurring episodes, (c) provide a distinct hands-on treatment which is tangible, immediate, eliciting the benefits of therapeutic touch [8], and which dramatically addresses the patients symptoms. Such an encounter provides multiple powerful contextual cues for patients, including expertise, authority, and confidence that the chiropractor understands and can manage the cause of the patient’s symptoms [9].

The connection between pain and movement that patients experience and the mechanical nature of SMT, together with an audible pop that accompanies some types of SMT, makes for specific cues in support of a coherent and meaningful explanatory framework and experience. Indeed, the *story* associated with the therapy itself, as told by the practitioner, has been shown to be independently effective for clinical outcomes [10]. This supports a strong therapeutic relationship and reinforces high expectations that a patient’s painful condition is likely to be mitigated. That patient expectations significantly predict clinical outcomes for pain is increasingly supported by evidence, both clinically and through the discovery of specific neurological mechanisms that modulate pain through expectation [11–14].

In this sense, the contextual framing of SMT is perhaps very different to that of physical exercises, cognitive therapy, and pharmacological treatment: The contextual frame of SMT is particular in being one of *direct individually curated action with an immediate response*. We suggest that many patients will point to this as the key reason for seeking out a chiropractor [15], if not in so many words. For that reason, if no other, we suspect SMT will and should continue to play a role in the future management of MSK disorders, but within a wider framework that recognizes it as one of several interventions that include management of the patients’ cognitions and emotions as well as their body.

### Chiropractic training in the MSK area

With so much attention afforded to SMT by the profession, it is unsurprising that chiropractic undergraduate education is primarily concerned with biomechanics and the MSK system. It would be reasonable to conclude that chiropractic undergraduate training, in the same vein as dentistry, is directed at a specific area of expertise from the outset. By contrast, the undergraduate training of medical doctors, nurses and physiotherapists has a broader focus [16, 17].

Clearly, non-MSK disorders constitute a significant part of the chiropractic curricula in relation to general and differential diagnosis, but this tends to be somewhat peripheral to chiropractic treatment: merely enabling triage to ensure appropriate clinical management of disorders which require referral. Such diagnostic competencies are essential for safe clinical practice but are not central to the chiropractic scope of practice as such, and this is reflected in the time allocated in undergraduate chiropractic training.

That is also the case for developing clinical skills, in the area of psycho-social issues and therapies, which still seems to constitute only a minor proportion of the training time overall in chiropractic curricula, despite the profession apparently embracing the bio-psycho-social model of MSK pain [18].

By contrast, although the technicalities of SMT are overemphasised, chiropractic undergraduate training does provide solid base-competencies for independent management of MSK disorders and, in some settings at least, we would argue that when comparing new graduates from different professions, young chiropractors are uniquely well qualified to assume responsibility for managing MSK disorders.

### SMT as a strength of chiropractic

As a consequence of the profession’s almost myopic focus on SMT, undergraduate training is comprehensive as far as MSK disorders go, and clinical practice is generally well-aligned with recommendations for MSK disorders, being conservative, low cost, and safe.

## Opportunities

### MSK disorders

To point out that MSK conditions are a massive health problem has become cliché, but it is nonetheless an indisputable fact [19, 20]. What is more, MSK conditions are often poorly managed with therapies of questionable effect and with the potential for severe complications and side effects [21–23]. We shall use LBP as the primary example:

Paracetamol and Non-Steroidal Anti-Inflammatory drugs have no convincing clinical effect on spinal pain over placebo [24–26]. Whilst opioids do appear more effective than placebo, the effect size is not large; the numbers-needed-to-treat is three times that of the numbers-needed-to-harm, and opioid use is associated with severe complications and potential for abuse and should not generally be recommended in the management of LBP [27, 28]. Corticosteroids are not commonly used and are associated with only slightly better outcomes in radicular LBP compared to placebo, and probably no better for other types of LBP [29]. Orthopedic surgery in general (not spine) has been found to be comparable to placebo [30] and, whilst there are no placebo controlled trials for spinal disk surgery, the effects compared to conservative treatment are not impressive or long lasting [31, 32]. For spinal fusion surgery, there are also no placebo controlled trials, and effects are similar to conservative care [33]. Several controlled trials do exist for vertebroplasty, which is found to be no better than placebo [34], and, in general, surgery is found to have large contextual effects, especially on pain [35]. For exercise therapy, the evidence demonstrates it to be safe but of limited effect size, and, furthermore, the specifics of the type of exercise seem not to matter– i.e., any exercise is better than none [36, 37]. The same holds true for SMT [38], which has only slightly better or similar effects on short, intermediate and long-term pain and function compared to other recommended treatments (e.g., exercise therapy) and non-recommended treatments (e.g., ultrasound and corsets). Further, there is no evidence that the type of SMT makes an important clinical difference.

Thus, although a veritable smorgasbord of treatments is available for patients with MSK disorders like LBP, and while some are more expensive or associated with higher risks, none has proven decisively superior to others. However, exercise and SMT stand out as being both safe and inexpensive.

Furthermore, the organization of healthcare services and the roles of healthcare providers matter. For instance, the catastrophic impact of the opioid crisis is well documented (see the Centers for Disease Control website for an overview [39]), the coverage policies of third-party payers affects patient behaviours and are important for healthcare utilization and outcomes [40, 41] and the risk of opioid use has been demonstrated to be lower with chiropractic care [42, 43].

### A new clinical role

In short, it is becoming increasingly clear that MSK conditions, such as LBP, will generally not be cured by drugs or surgery, as early chiropractors predicted. However, neither will they be cured by SMT, exercise, or other known conservative approaches. Instead, as such conditions are often recurring and fluctuating, they need to be managed and self-managed safely and rationally in the *long-term*. Each of the treatments currently available may have a role to play in that long-term management, but none of them, including SMT, have proven decisively superior to the others and clinicians therefor need a *broad-focus* armamentarium to choose from.

This suggests a need for *someone* (a healthcare professional) rather than *something* (a treatment) to play a pivotal role for long-term management. That *someone* should assume a central and directing role in a collaborative interdisciplinary setup [44]. We consider chiropractors well suited for such a role, at least in some countries and settings, and adapting chiropractic practice to the reality of MSK disorders, as it has been uncovered by growing scientific evidence, constitutes a window of opportunity for the profession.

### Future roles for chiropractors

Chiropractors’ specialized pre-graduate training with particular emphasis on MSK disorders, a tradition for conservative clinical management together with close contact with their patients, often well-developed interpersonal skills such as clinical relationship building, and high availability already single them out as ideal clinical players in this area of healthcare. Nevertheless, it is a role which will require a particular emphasis on new and presently de-prioritized skills.

Near the top of the list of new priorities is the ability to work in collaboration with other professionals, based on extensive knowledge of all the clinical facets relevant to the long-term management of MSK disorders, a knowledge which presently is patchy. This includes appropriate use of the range of evidenced interventions and mediating contextual factors that are available, an ability to judge when it would be appropriate to secure timely referral for specialist evaluation (requiring relevant referral networks), an insight into how to support self-management and to avoid harmful illness behaviour, and knowledge and skills making it possible to support high levels of functioning and employment. In addition, familiarity with the complex workings of national healthcare systems, multidisciplinary healthcare settings, and networks will be necessary. All this would require additional training and certifications. In many ways, such a role is diametrically opposite to a primary care solo practice where the main focus of examination is to *find it* and the mainstay of treatment is to *fix it* with SMT, and the response to *failure* is to adapt the SMT technique and try again.

Against this background, technical eminence in the application of SMT will not suffice anymore if the profession’s aspirations are to talk to and work with anyone other than their patients. That is central to avoiding becoming increasingly isolated and marginalized from the wider healthcare landscape. In turn, it will require effective and coordinated collaboration with, not only other health-care professionals, but also employers, social workers, public authorities, third party payers, and other relevant stakeholders.

This imperative is more acute in some national jurisdictions than others. For example, osteopaths in the UK are already part of a governmentally recognized group of healthcare professions, the Allied Health Professions. This group enjoys integration with or opportunities for integration within the wider healthcare system along with career progression and skills development funded by government sources. Presently, chiropractors remain the only regulated profession in the UK absent from this group, with all the ensuing barriers to accessing resources, professional progression, and public recognition that the exclusion from such cultural legitimization might bring [45].

It is clear that a central directing role, such as we have described it, will prove a very different role from the one most chiropractors assume today, and getting there will necessitate willingness to adapt and embrace new perspectives from an otherwise very conservative profession.

It will also define a new role for SMT within chiropractic: from an identity-defining paradigm to *simply* one of many tools in a much larger armamentarium within a comprehensive package of care. Further, it requires the acknowledgement that this wider armamentarium is not the sole responsibility of the chiropractor but that it is to be provided by a group of collaborating practitioners. Such a change is not given and far from trivial, albeit essential. As we see it, three paths now lie open for the future of the profession;

Path 1: Chiropractic could re-invent itself with some urgency, with less focus on the role of SMT and shift away from a chiropractic identity as providers of SMT within a distinct theoretical framework, towards a broader role as coordinators of long-term management of MSK disorders well-integrated in the wider health-care landscape [A role as an MSK manager].

Path 2: Chiropractic could give up any ambitions of better mainstream integration in the healthcare system/market and embrace an identity as unambiguously alternative on par with naturopathy, homeopathy, reflexology, etc. [A role as alternative fringe practitioners].

Path 3: Chiropractic could accept a limited albeit mainstream role as manual therapists on par with several other professions in a setting, where SMT is prescribed as a delegated task in the long-term management of MSK, directed and coordinated by someone else [A role as a manual therapist].

Either one of these alone represents a potential future path for the profession, but all three at the same time do not. Conversely, these paths are not equally realizable in all countries and cultural settings.

## Where to next and how to get there?

It has been said before that chiropractic is at crossroads [46, 47]. The future, which ever direction it takes, may be forced upon us, but to avoid this, the chiropractic profession needs to set its own heading for the future and to move in that direction as a unified profession. The alternative is to seriously consider whether breaking up into different professions is, in fact, the way forward [48]. In either case, we are convinced that the status quo is untenable.

We admit to being somewhat pessimistic that the chiropractic profession is actually able to unify and change to assume the role we have outlined, and we suspect that the window of opportunity to do so is already closing rapidly. In some parts of the world, it is firmly shut already.

Nonetheless, if the profession is to shift away from the role of primarily providers of SMT, towards a broader role as central MSK coordinators (i.e. Path 1 in the above), we suggest the following steps need to be taken as soon as possible.

### Chiropractic professional organizations

We suggest that all chiropractic organizations need to carefully consider and ultimately include in their organizational vision statements a clear stance on the future of the profession in relation to the following points:

- The current professional heterogeneity (e.g. “*philosophy*”, scope of practice, identity) is by its very nature divisive and thus detrimental to progress.
- Maintaining a position that seeks to ignore or appease traditionalists who refuse to move forward for the sake of perceived political or financial status quos will be seen by future generations of chiropractors as merely hastening the decent of the profession into irrelevancy.
- The current situation cannot be ignored anymore but must be dealt with resolutely and with urgency. Not to make such a decision is also a decision, but one that leaves the outcome to chance or in the hands of others.
- The profession needs to decide, *embrace* and communicate that its raison d’être is not anchored to SMT– the centrality of *SMT* in professional identity must be replaced with an actual *evidence-based scope of practice*.
- The profession needs to declare unequivocally that it has a focused and limited scope of practice with MSK disorders at its center.
- The profession takes it upon itself to become self-evidently specialized in and competent beyond reproach to manage a broad range of healthcare services related to MSK disorders, and the profession abandons the identity of primarily providers of manual therapy, by whatever name.
- The profession needs to ensure that this is communicated effectively and un-ambivalently to chiropractors themselves, the public, other healthcare providers, politicians, third-party payers, employers, etc.
- The profession needs to ensure that arrangements regarding reimbursement, insurance, and legal status are aligned with such a scope of practice, e.g. reforming reimbursement for ‘correcting vertebral subluxations’ in the USA.

Importantly, the profession needs to decide how to ensure or enforce such a development.

### Chiropractic teaching institutions

The students of today are not just the chiropractors of tomorrow. They are also the professional leaders, influencers, teachers, researchers and role-models of tomorrow. If progress can be wrought, this is were it will most likely come from. We therefore, suggest that all chiropractic accrediting authorities and teaching institutions must carefully consider their pivotal role in long-term professional change and that their organizational vision statements reflect the points raised above.

- Teaching institutions must provide education that is evidence-based and rooted in clinical science rather than dogma-based rooted in the “*art, science and philosophy*” of chiropractic.
- Teaching institutions must prioritize the development of critical thinking skills through increased emphasis on the history, philosophy, and practice of science and how it relates to healthcare, including how to identify clinical pseudo-science.
- Teaching institutions must make it their primary goal to ensure that their students acquire all the clinical competences, which scientific evidence suggest is relevant to the management of MSK disorders irrespective of its current place in chiropractic history and dogma.
- Teaching institutions must review and rapidly move toward changing the SMT-centric culture carried over from a century ago and replace it with the rational and critical approach of a progressive profession, including long-term management of patients with MSK disorders.
- Teaching institutions must cease any direct or tacit support of the idea of SMT-exceptionalism and instead treat SMT as *one* relevant tool among many equals within a package of clinical skills. This should entail embracing SMT as a non-specific contextualized intervention and dialing down the preoccupation with manual technique details, instead fostering realistic expectations for treatment outcomes, especially in chronic pain.
- Teaching institutions must spend less time on the technicalities of SMT, which is evidently likely to be of little clinical consequence. Instead, they should use that freed time to improve education in the other evidenced clinical skills needed to manage MSK disorders. In particular, they should put increased emphasis on addressing psychosocial aspects of care, such as assessing beliefs and fear of pain. This includes building effective and ethical therapeutic alliances with patients [49–52] and constructive communication and cooperation with other professionals.

As an encouraging example of efforts to effect real professional development, we direct the reader’s attention to the position and implementation statement of the International Chiropractic Educational Collaboration [53].

### Public chiropractic opinion makers

The pre-occupation with SMT is also evident on social media, where chiropractors can reach a large audience, particularly younger individuals. Much time and effort are also afforded to different ongoing education courses, where the minutiae of SMT techniques take center stage, often accompanied by some form of theoretical framework to provide a rationale for the technique in question.

With such *influencers*, public opinion-makers, youtubers, tiktokers, and technique gurus in mind, we suggest:

- That influencers acknowledge that they are just that: ‘*influencers*’. As such, their responsibilities extend beyond those of any individual chiropractor and they should exemplify those of an ambassador of the profession.
- That influencers advocate patient-centred, professional and evidence-based MSK care that stays within the relevant scope of practice, including support for self-management strategies.
- That influencers stop presenting SMT as an exceptional tool, required to optimize health or promote a life of maximum potential, and instead depict it as a skill among other clinical skills that can be used within a larger management strategy for MSK disorders only.
- That influencers accept that a case-report carries no real scientific weight and should not be presented as evidence. Case-reports are impactful tools in communicating with the public, and influencers should, therefore, use them carefully and only to illustrate that which is supported by actual evidence.
- That influencers be very careful not to auction off their professional responsibilities in a popularity contest of hits, clicks and likes, but rather use their popularity as a platform to disseminate sound patient education to the public [54].

### Individual chiropractors

What can the individual chiropractors do? Chiropractors need to be ready to radically update their clinical practice and communication with patients, the public, and especially other healthcare stakeholders as and when appropriate. We ask individual chiropractors to embrace the following.

- That chiropractors once-and-for-all join the post-enlightenment revolution and, with it, the recognition that *actual* knowledge is continuously expanding and improving through the process of science and rationality, and not something handed down as tenets from a historical authority.
- That chiropractors reject doctrine and instead adopt a stance of readiness to change their clinical practice in the face of evolving knowledge.
- That chiropractors should restrict themselves to MSK disorders and take pride in safely and effectively managing these global priority conditions, rather than chasing poorly defined and unmeasurable concepts like ‘a life of maximum potential’ or disorders which they are not educationally or clinically equipped to manage.
- That chiropractors acknowledge and accept that there is no rational foundation for SMT-exceptionalism and instead commit to implementing a broader clinical armamentarium. In doing so, they should afford less time and energy on SMT technicalities, in general, and place more emphasis on the broader management skills in MSK disorders, e.g., as outlined elsewhere in the seven roles of health-care professionals [55].

## Conclusion

We have argued in this and a previous paper that for any real professional development to take place, it is essential that chiropractors work specifically with their understanding of the role of SMT in chiropractic identity. We have outlined some strengths, weaknesses, opportunities and threats posed by SMT in relation to the future of chiropractic.

We have argued that the fracture line that splits the chiropractic profession stems from fundamentally different perspectives of SMT and the historical dogmas versus contemporary evidence that inform its use. It is a foundational fracture that requires continuous efforts to paper over and which has become an existential threat to the profession: While parts of the profession subscribe to EBM and seek greater integration, other parts are marching backwards toward 1895 under the banner of vitalism, which to quote Simpson and Young “*sits at the heart of the divisions within chiropractic and acts as an impediment to chiropractic legitimacy, cultural authority and integration into mainstream health-care*” [56].

As the adage goes, the definition of insanity is “*doing the same thing over and over again, expecting different results*”. We contend that it is time for chiropractic to do something new: Commit to one side of the fracture line or the other and do so in unison. We clearly recommend the side aligned with EBM and have made specific recommendations in that regard for chiropractic organizations, teaching institutions, influencers, and practitioners.

## Data Availability

Not applicable.

## Abbreviations

SMT: Spinal manipulative therapy
MSK: Musculoskeletal
